# WHONDRS: a Community Resource for Studying Dynamic River Corridors

**DOI:** 10.1128/mSystems.00151-18

**Published:** 2018-10-09

**Authors:** James C. Stegen, Amy E. Goldman

**Affiliations:** a Ecosystem Sciences Team, Pacific Northwest National Laboratory, Richland, Washington, USA; b Ecology Group, Pacific Northwest National Laboratory, Richland, Washington, USA

**Keywords:** biogeochemistry, hydrology, hyporheic, microbial ecology, microbiology, research consortium, river

## Abstract

The Worldwide Hydrobiogeochemistry Observation Network for Dynamic River Systems (WHONDRS) aims to galvanize a global community to provide the scientific basis for improved management of dynamic river corridors. WHONDRS is a global research consortium working to understand connections among dynamic hydrology, biogeochemistry, and microbiology in river corridors from local to global scales. WHONDRS ascribes to the perspective that resources, knowledge, and data belong to the community as a whole and that science advances more rapidly and more robustly through community ownership. As such, WHONDRS provides free access to novel instrumentation, molecular analysis, and well-curated data associated with river corridor hydrology, biogeochemistry, and microbiology. There are a number of ways to be involved in WHONDRS, ranging from one-time surface water sampling to installation of WHONDRS-developed multiparameter sensors for continuous monitoring. WHONDRS hinges on broad involvement, and we encourage all interested parties to contact us and become part of the consortium.

## Abstract (precis)

The Worldwide Hydrobiogeochemistry Observation Network for Dynamic River Systems (WHONDRS) aims to galvanize a global community to provide the scientific basis for improved management of dynamic river corridors. WHONDRS is a global research consortium working to understand connections among dynamic hydrology, biogeochemistry, and microbiology in river corridors from local to global scales.

## EDITORIAL

River corridors integrate surface water, the subsurface, as well as the land and vegetation surrounding rivers, and subsequently provide a broad range of services to society (e.g., improved water quality and habitat for myriad taxa) that arise through interactions among biological, physical, and chemical processes. These interactions revolve around hydrologic connections, including inputs from precipitation, runoff, and groundwater, which lead to dynamic and highly coupled systems. To facilitate broad understanding of these critical systems, we launched the Worldwide Hydrobiogeochemistry Observation Network for Dynamic River Systems (WHONDRS).

Led by a team at the Pacific Northwest National Laboratory (PNNL), WHONDRS is a global research consortium working to understand connections among dynamic hydrology, biogeochemistry, and microbiology in river corridors from local to global scales. WHONDRS aims to galvanize a global community to provide the scientific basis for improved management of dynamic river corridors. This is achieved by providing free access to novel instrumentation, molecular analysis, and well-curated data that will be hosted on the ESS-DIVE data archive (https://ess-dive.lbl.gov/). ESS-DIVE is a broad archive for all data generated by projects associated with the U.S. Department of Energy’s environmental research efforts within the Biological and Environmental Research program. Efforts are under way to move WHONDRS data to ESS-DIVE and enhance the discoverability and use of these data.

WHONDRS ascribes to the perspective that resources, knowledge, and data belong to the community as a whole and that science advances more rapidly and more robustly through community ownership. As such, the scientific community is encouraged to use WHONDRS data—with no strings attached—in their analyses and/or to perform secondary reanalysis to pursue additional science questions, evaluate the reproducibility of previous analyses, and deepen understanding with future improvements to data analysis approaches. A strength of having WHONDRS data freely available to all and within a consistent format is that anyone will be able to easily take the data and use them to advance science in new and innovative ways with no restrictions or obligations beyond acknowledging the data source.

WHONDRS works with the community toward broad characterization that can facilitate generalizable understanding of river corridors. Key to the transition from characterization to understanding is the application of consistent methods across a wide range of river corridor systems. Generating data in a consistent manner across systems offers opportunities for both discovery-based and hypothesis-driven efforts. For example, in discovery mode, WHONDRS data will be interrogated with machine learning tools to draw out common features (e.g., metabolites with particular features related to their biogeochemical role) or relationships (e.g., associations between certain groups of microbial taxa and multivariate profiles of biochemical transformations) that are found across all systems or within particular spatial domains or environmental contexts. As a complementary approach, some field sampling campaigns will be designed to target particular hypotheses and science questions, such as the hypothesis that smaller river corridors will show greater temporal coherence between surface and subsurface metabolite dynamics. As discussed below, there are three WHONDRS efforts under way meant to enable both discovery- and hypothesis-based learning: (i) a global survey of surface water metabolites, (ii) high temporal resolution of metabolites and microbiomes in surface and pore water, and (iii) hydrologic exchange and redox chemistry.

## 

### Global survey of surface water metabolites and microbiomes.

The global survey of surface water metabolites and microbiomes examines the global biogeography of metabolites to provide understanding of the character of organic carbon that may be delivered to subsurface sediments via hydrologic exchange. This study focuses on a variety of interrelated science questions, such as the following questions. Is a core metabolome shared across all streams? Are there aspects of coupled hydrologic and biogeochemical dynamics that consistently drive metabolite profiles across systems? Is there enough spatial and temporal consistency in the drivers of surface water metabolites to generate a global atlas of these features?

**FIG 1:**
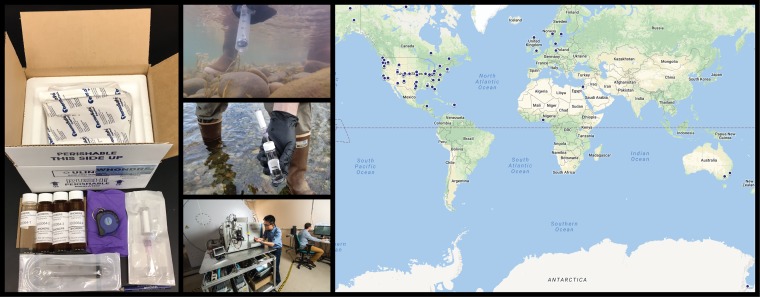
(Left) WHONDRS sampling kit that is sent to collaborators around the world for surface water sampling. Samples are collected using a syringe, passed through a 0.22-μm filter into glass vials, and shipped overnight to PNNL/EMSL on blue ice in the pictured cooler/box. Additional pictures show use of the sampling kit for in-water syringe-based water collection, filtration of the water sample, and an FTICR-MS instrument within EMSL used to characterize organic metabolites within filtered water samples. (Right) The map shows locations of current or planned WHONDRS surface water sampling sites (blue circles). The number of sites is increasing rapidly, along with the spatial coverage and types of streams and rivers being studied. WHONDRS is free to participate in, and we encourage interested parties to contact us through email (WHONDRS@pnnl.gov) or social media (@WHONDRS) and to learn more on our website (https://whondrs.pnnl.gov). Background map courtesy of Google Maps.

To implement the global survey, a free stream sampling kit is being provided to interested researchers throughout the world (Fig. 1). Samples are collected with minimal constraints (see the WHONDRS YouTube channel [https://www.youtube.com/channel/UC8d9IFF3qMRkJlo2SlWAz3Q] and website [https://whondrs.pnnl.gov/] for details) and shipped to the Environmental Molecular Sciences Laboratory (EMSL; https://www.emsl.pnl.gov/emslweb/) for metabolomic analysis via Fourier transform ion cyclotron resonance mass spectrometry (FTICR-MS). In addition, basic geochemistry analyses (e.g., dissolved organic carbon concentration) are conducted, and all materials and analytical costs are covered by PNNL via funding from the U.S. Department of Energy’s Subsurface Biogeochemical Research program. Through a collaboration with Kelly Wrighton at Colorado State University, this effort is also being extended to microbiome analyses (i.e., amplicon, metagenomic, and transcriptomic sequencing) on the same samples used for metabolomics.

### Time series of metabolites and microbiomes in surface and pore water.

The time series of metabolites and microbiomes in surface and pore water investigates coupled temporal fluctuations in both surface water and subsurface pore water metabolite profiles and microbiome composition in systems that experience sustained, high-frequency stage (river depth) fluctuations. In this case, high frequency refers to daily or subdaily fluctuations, and sustained indicates that these fluctuations occur throughout a significant portion of the year or growing season. There are a number of factors that result in such fluctuations: (i) the operation of hydroelectric dams, (ii) tides, (iii) evapotranspiration from vegetation, (iv) wastewater discharge, (v) water extraction for human use, (vi) glacial melt, and (vii) wind-driven fluctuations in very large lakes. This study will first look at one example system associated with each of these seven driving mechanisms of high-frequency fluctuations. WHONDRS members will collect time series samples in each system across multiple cycles of stage fluctuations. Metabolomics, microbiome, and geochemical analyses will be conducted. Resulting data will become immediately public, and costs for materials and analyses will be covered by WHONDRS.

### Hydrologic exchange and redox chemistry.

Exchanges of water between a river and the surrounding sediments—and the biogeochemical processes in those sediments—are critical to the health and function of river corridor systems. The highly dynamic nature of these systems poses significant challenges to measurement of hydrologic exchange and associated biogeochemical responses. To meet this challenge, WHONDRS is developing sensor technology that will be freely distributed to WHONDRS collaborators (see the WHONDRS YouTube channel for an overview [https://www.youtube.com/channel/UC8d9IFF3qMRkJlo2SlWAz3Q]). The sensing system estimates multiple parameters within the riverbed across a vertical profile across dynamic flow conditions. WHONDRS is collaborating with EMSL computational facilities to enable WHONDRS members to easily (and freely) model the resulting data using previously developed computational code run on the EMSL supercomputer.

### Contribute to a global community effort.

We invite all interested parties to join WHONDRS and note that there are a variety of benefits to becoming a WHONDRS member, such as the following:

- Joining a global effort to understand the holistic functioning of dynamic river corridors.
- Accessing high-end infrastructure and methods such as ultrahigh resolution metabolomics, microbiome analysis, and novel sensing technology.
- Generating data and knowledge that can be leveraged towards more detailed system-specific investigation, while also contributing to global understanding.

WHONDRS hinges on broad involvement, and we encourage interested parties to contact us through email (WHONDRS@pnnl.gov) or social media (@WHONDRS) and learn more on our website (https://whondrs.pnnl.gov). In addition to engaging individual researchers, WHONDRS is working to leverage existing efforts such as the U.S. Geologic Survey’s stream gauging program, the National Ecological Observatory Network, and international efforts such as the intermittent rivers project. In addition, WHONDRS is conforming to existing data standards and working to connect generated data with existing repositories, such as the Earth Microbiome Project. By pairing with individual researchers and existing programs, WHONDRS aims to advance our collective knowledge of river corridors so that these critical systems can sustainably interact with human society for many generations to come.

